# Implementing delayed umbilical cord clamping in Nepal—Delivery care staff’s perceptions and attitudes towards changes in practice

**DOI:** 10.1371/journal.pone.0218031

**Published:** 2019-06-12

**Authors:** Nisha Rana, Olivia Brunell, Mats Målqvist

**Affiliations:** International Maternal and Child Health (IMCH), Department of Women’s and Children’s Health, Uppsala University, Uppsala, Sweden; Cincinnati Children’s Hospital Medical Center, UNITED STATES

## Abstract

**Aim:**

To explore delivery care staff’s perceptions and attitudes towards changes in practice of umbilical cord clamping in order to identify work culture barriers and enablers for improved clinical practice and implementation of the new guidelines on cord clamping.

**Method:**

A purposive sampling strategy was used to include delivery staff at two major hospitals in Kathmandu, Nepal for focus group discussions. Key informant interviews were conducted with the ward in-charges and skilled birth attendant trainers at the respective hospitals. Data were analyzed using qualitative content analysis.

**Result:**

Eight focus group discussions altogether with 34 delivery care staff working in the labor room and birthing units, and 12 key informant interviews with skilled birth attendant trainers/supervisors and ward in-charges from both hospitals participated in the study. Participants had positive attitudes towards delayed cord clamping as it was not perceived to be a difficult task and as they perceived it to be beneficial for mother and child. The “will to do good”, and a high level of trust both in the hierarchical system as well as in scientific evidence were identified as promoters of change. Several barriers were mentioned, such as maternal or fetal medical conditions and physical settings, as constraints to perform delayed cord clamping. They also mentioned difficulties in adopting new guidelines due to habitual practice, lack of formal training and poor coherence within the work team. In order to bring change to the practice, participants highlighted that officially approved national and institutional protocols and regular training are crucial.

**Conclusion:**

Due to poor coherence within the health system and lack of national or institutional protocols, delivery staff has to rely on their own initiative to develop skills and are left to informal decision making, and are therefore hesitant to apply new routines. In order to change cord clamping practices to align with evidence and policies health-care staff needs to be better supported by the governance structures of the health system, with clear and approved guidelines made available and coherent training and support.

## Introduction

Clamping and cutting the umbilical cord after birth is one of the steps in the birth process. Debate about the appropriate time to clamp and cut the umbilical cord has been ongoing for many decades [1]. The use of early cord clamping (ECC), which is generally carried out during the first 60 seconds after birth (most commonly in the first 15–30 seconds) has been common practice in delivery care around the world [1]. However, recent research has demonstrated multiple benefits of waiting for more than 60 seconds to cut the cord [2–8] and WHO guidelines have recently been changed. The current recommendation is to practice delayed cord clamping (DCC), i.e. to wait for at least 60 seconds after birth before clamping the cord [1].

Despite the published benefits to infant and mother of delaying umbilical cord clamping, there is little evidence that this practice routinely occurs in hospital settings [5]. In routine practice, health-care providers tend to cord clamp either as they were clinically trained or as per organizational trend where they work [5]. Immediate or early clamping and cutting of the umbilical cord is thus common practice among health-care providers. Transitioning to new routines takes time and patience [9], since health-care workers normally continue to practice in the manner in which they are familiar. There could also be additional resistance to DCC when the baby is born prematurely or when babies need resuscitation after delivery [5].

In 2014, we conducted a cord clamping study at a tertiary hospital in Nepal, the Paropakar Maternity and Women’s Hospital (PMWH), to investigate the effect of DCC after 180 seconds versus ECC within 60 seconds [10]. In this study, we found a high incidence of protocol deviation in the DCC group where 22.6% of the infants in the delayed group underwent cord clamping before 60 seconds since the nurse midwives perceived that early clamping against the allocation was required [2]. Other studies note that it is feasible to introduce DCC even if uptake is slow and requires training and refreshers [11]. There are also examples of obstetric units where delayed clamping is routinely and successfully applied also among distressed infants, demonstrating the feasibility of delayed clamping in clinical practice [12]. In Nepal there are currently no clinical guidelines on cord clamping available, but the practice of delayed cord clamping is taught in skilled birth attendant (SBA) training. The objective of this study was to explore delivery care staff’s perceptions and attitudes towards changes in practice of umbilical cord clamping, with the aim to identify work culture barriers and enablers for implementation of the new guidelines on cord clamping.

## Methods

### Study settings

The study was carried out at two large referral hospitals in Kathmandu, Nepal. PMWH is a publicly funded maternity hospital for gynecological and obstetrics services with around 20,000 deliveries per year. It serves as a central referral hospital and training site for reproductive health, and one of the training centers for SBA. It has two separate delivery units, a labor room for high-risk deliveries and Maternal and Neonatal Service Center (MNSC) for low-risk deliveries, also called the ‘birthing unit’. Tribhuvan University Teaching Hospital (TUTH) is a non-profitable hospital with about 7,500 deliveries per year. It is a central referral hospital which deals with all types of medical services, including maternity services. The hospital has two distinct functions, as a teaching hospital and as a national hospital. The hospital is also one of the SBA training centers. This hospital has two delivery units, a high-risk labor room and a low risk birthing center. Birthing centers at both hospitals are managed by SBA trained nursing staff, except in the case of unexpected emergencies when extra staff may be brought in.

### Design and participants

To explore delivery care staff’s perceptions and attitudes towards changes in the practice of umbilical cord clamping, focus group discussion (FGD) and key informant interview (KII) were selected as preferred methods to collect data for analysis. The FGD and KII guidelines were developed with the participation and consensus of all the authors.

The nursing staff working in delivery units were invited to form groups to discuss the topic: *Delivery care staff’s perceptions and attitudes towards changes in the practice of umbilical cord clamping*. A purposive sampling strategy was used based on the following selection criteria: delivery staff involved in conducting deliveries in the labor room and birthing unit (MNSC) at PMWH; and in the labor room and birthing center at TUTH for FGD. Only nurse midwives who were on duty were available to participate in the discussion. Invitations were channeled through the ward in-charge at each delivery unit and arranged to fit the duty schedule. The ward in-charges and SBA trainers who were supervisors for the respective units were explicitly asked not to be present during discussions. The ward in-charges and SBA trainers were invited to separate KIIs. This was considered to be a suitable way to include perceptions at the managerial level and to increase variation of data. The characteristics of the participants are described in Table 1.

**Table 1:** Characteristics of the participants of the FGD and KII from PMWH and TUTH.

	Participants (n)	Years of experience^a^ Range (Median)	Participants with SBA training (n)	Degree attained^b^ (n)
**Key Informant Interview**				
**SBA trainers/supervisors**	8	19–33 years (28)	8	BN (6), MN (1), M.HCM (1)
**Ward in-charges**	4	25–28 years (27.5)	4	BN (4)
**Focus Group Discussion**				
**Labor room (TUTH)**	14	4 months-16 years (6)	2	PCL (2), BN (6), BSc Nursing (5), MN (1)
**Birthing Center**	4	9–22 years (15.5)	3	BN (3), BSc Nursing (1)
**Labor room (PMWH)**	6	8–17 years (14.5)	6	BN (6)
**Birthing Unit**	10	5–22 years (12.5)	9	PCL (2), BN (8)

^a^ Year of experience within health care among participants

^b^ Degree attained: PCL = Proficiency Certificate Level in Nursing, BN = Bachelor in Nursing, BSc Nursing: Bachelor science in Nursing, MN = Masters of nursing, M.HCM = Masters in Health Care Management,

### Data collection

To generate a trustworthy environment the first author visited both hospitals and described the purpose of the study. Invitations to participate in the study were channeled through the ward in-charge at each delivery unit at the two hospitals. Arrangements were made so that all nursing staff had the possibility to participate. The first author moderated all the FGDs and KIIs in Nepali. Four groups of delivery care nurses working in the labor room and birthing units from each hospital, 8 groups in total, were included in FGDs with 3–5 participants in each group. Three KIIs from TUTH and 9 KIIs from PMWH were conducted with supervisors (ward in-charges) and SBA trainers/supervisors. The FGDs and KIIs lasted between 20 and 40 minutes. For the FGDs, the number of participants needed to be large enough to generate a meaningful group discussion but small not to interfere with the different duty schedules.

Before analysis, both FGDs and KIIs were recorded by voice recorder, transcribed verbatim in the local language (Nepali) and translated into English by the first author.

### Data analysis

An inductive approach to data was applied and analysis was performed using qualitative content analysis as outlined by Graneheim and Lundman [13]. Transcripts were coded manually and after coding text segments, codes were condensed into sub-categories which were given descriptions reflecting the content.

The next stage of analysis was performed by all the authors together. Working together in a back-and-forth process, the subcategories were sorted into categories. To increase the descriptive value of the categories, some level of abstraction was used, but the authors still attempted to accurately reflect the content of the subcategories.

The first author then went back to the original text to find quotes that were representative of each category. The categories and subcategories and related quotes were finally presented to all co-authors to allow them to comment and agree on the findings.

### Validity and reliability of tools

The instrument for data collection in this study was an interview guide with open-ended questions developed after extensive review of the literature. The tool was reviewed by the last author to check for face and content validity and translated and back-translated to and from Nepali to English.

### Ethical considerations

Ethical clearance was obtained from the institutional review board of both hospitals; Paropakar Maternal and Women’s Hospital (58-5-1561) and Tribhuwan University Teaching Hospital (date: 2018-03-11) and from the Nepal Health Research Council (Reg.no:17/2018) on 15 April 2018 before data collection started. All participants signed a written informed consent form before the discussion started and were reassured that the confidentiality of the information would be respected. The participants were informed about the possibility to withdraw from the study at any time without any sanctions or required explanation. The study participants were at no risk of harming themselves or others by taking part in this study. Participation in the study was voluntary. Notes and audio records were kept in a safe place by the moderator. The moderator transcribed in Nepali and translated it into English. The participants’ anonymity was maintained throughout data collection, analysis and reporting.

## Results

The inductive analysis process generated six categories and 18 sub-categories, presented in Table 2.

**Table 2:** The categories and their corresponding subcategories from both FGDs and KIIs.

Categories	Sub-categories
**Medical and physical barriers**	Maternal medical condition
	Fetal medical condition
	Physical setting
**Habitual practice**	Routine work
	Need for reminders
	Need to have knowledge about benefits
	Change through practice
**Forced to informal learning**	Lack of refresher training
	Peer learning, and informal information
**Lack of coherence**	Lack of team work
	Lack of trust among team
**Need to bring uniformity**	Knowledge update through coaching and orientation
	Request for authorized protocol
	Supervision, monitoring and evaluation
	Use flex chart, poster and pamphlet to raise awareness
**Opportunities for change**	The will to do good
	Research as authority
	Trust in hierarchical system

### Medical and physical barriers

Participants highlighted maternal or fetal medical conditions as a constraint to perform DCC. Conditions such as maternal distress, complicated labor, pre-eclampsia, postpartum hemorrhage and many more were cited as making it difficult to wait to clamp the cord. The informants also claimed that sometimes the mother was not ready to keep the newborn on her abdomen, either because she was uncomfortable or afraid, or just not cooperative. At times like this, the staff perceived that they needed to cut the cord immediately and separate the newborn from the mother.

“some mothers might not be prepared to keep their baby on their abdomen”

(Matron)

“some mothers would get afraid and say don’t keep it on my abdomen…they would say “I don’t want to hold the baby”.”

(Birthing center in-charge)

Nor was it perceived as possible to wait for DCC when the baby was floppy, asphyxiated or not crying, had thick meconium staining, cord tightly around the neck, short cord, or if the baby has septicemia.

Besides maternal and fetal conditions, physical conditions were highlighted as barriers to DCC. In the operating theater the delivery bed is small and it is difficult to keep the baby on the mother’s abdomen, the room temperature is low especially in winter, and sometimes due to lack of manpower and heavy work load delivery staff cannot wait for DCC.

### Habitual practice

Participants mentioned difficulties in adopting new guidelines due to habitual practice. They get stressed and feel the need to cut the cord before any other procedure as they are used to. It was emphasized that it is more difficult for experienced people to adopt new routines as they have been carrying out old practices for a long time.

“Very senior sisters, they themselves don’t want to change, they want to do what they have been practicing.”

(SBA trainer/supervisor)

“They tend to do what they have been doing in the past.”

(SBA trainer/supervisor)

The participants also thought that as delivery staff is busy with routine work, they forget the new practice, and that they require reminders more frequently at the beginning. Reminders, especially from ward in-charges and supervisors, were raised as being important.

“They remember only after we remind them about delaying the cord clamping”

(Ward in-charge)

The participants believed that all delivery staff should have knowledge about the benefits and contraindications of DCC, and they mentioned the need for coaching or training. Good knowledge was perceived as necessary for self-motivation, which in turn was highlighted as essential to adopt change.

“We should tell them about the benefits of it. If we could make this clear to them then they would start doing it and they could also do it”

(SBA trainer/supervisor)

“More than that, it should come from their inner heart, from their attitude”

(SBA trainer/supervisor)

The participants stated that change can take place through regular practice. They thought that there should be a demonstration at the beginning and that they should be supported in practicing regularly until it becomes a new habit.

“It’s all about practice and if they get into practice then it continues.”

(SBA trainer/supervisor)

“There should be simulation and at the beginning they should have supervisors with them, that will help them to learn it gradually”

(Nurse midwife)

### Forced into informal learning

Participants stated that they are left to rely on informal learning as there are no refresher training opportunities for SBA trained staff nor are there any knowledge updates about DCC. Instead, they felt they were forced to get their information from peer learning, self-education and in-service learning. Since there is no formal education about DCC they need to take their own initiative to learn new ideas, and the participants claimed that they have to rely on informal information.

“We had SBA training 2–3 years back, we don’t have training that often so… if it was frequent then we would have been updated too but there is not much training, that’s why”

(Ward in-charge)

“Delayed cord clamping had been discussed in the ward and I also have searched on the Internet, on the NCBI site, I have searched on it”

(Nurse midwife)

“…like it says on Facebook also, like it says “pregnant mother is beautiful”. There as well, it talks about delayed cord clamping so we started it… from that they changed in the curriculum so we also started doing it”

(SBA trainer/supervisor)

### Lack of coherence

Participants experienced a lack of coherence within the team. Not only a lack of teamwork but also a lack of trust among team members was perceived to hamper the efforts to bring uniformity.

“It would have helped to have the same thoughts because even though we are a different team, our goal is the same, only our way is different…. patient comfort, condition of patient, safe mother and child…. It would have been better if that could have happened”

(SBA trainer/supervisor)

Nursing staff expressed that they thought that the medical doctors feel they are superior to them and that they don’t have to follow what nurses say, even though it is the latest information that the doctors are unaware of.

“If doctors are involved in a delivery then they ask, “Who said delayed cord clamping?”

(Nurse midwife)

“Yes, they feel superior… ‘we are doctors and you are just a nurse. Why should we listen to nurses’? We feel it, we have the same value as they have but they feel that, ‘nurses are our assistants’.”

(SBA trainer/supervisor)

### Need “to bring uniformity”

The participants stressed the need for uniformity in clinical practice. This should be achieved through knowledge updates through onsite coaching, refresher training for those who have already received SBA training and orientation to all delivery staff nationwide. DCC should be included in the nursing curriculum and SBA training manual and thus be formalized.

“Those who are exposed to conducting delivery should formally know about it. Small orientations or one- or two-day packages as per content volume.”

(Nurse midwife)

“It should be included in the SBA course. Secondly, it should be included in the bachelor’s and master’s curriculum and after that every hospitals and periphery level should provide orientation.”

(Ward in-charge)

The participants also requested a standardized protocol at the national level and also within the hospital. Protocols guide and put pressure on delivery staff to follow and adopt new guidelines. It was emphasized that new protocols should be disseminated through official written documents or letters so that they will reach each and every part of the country. It was perceived that this would help to spread the information quickly and in an authorized way.

“It would be best if we could make it a protocol, if this is possible. It would be useful for ever, everyone would read it and apply it. It would go to every place and everyone would do their work as per the protocol.”

(SBA trainer/supervisor)

“There should be authorized instructions and then everyone would do it.”

(Nurse midwife)

“Until and unless it comes as a national protocol, no one will do it.”

(SBA trainer/supervisor)

The participants recommended including DCC in the partograph and having regular supervision and monitoring from a higher level. It was also suggested that there should be a monitoring body to check if change has taken place as per the protocol, since it is difficult to bring about change without effective supervision and monitoring.

“It might also help if we were to make a habit of noting the timing of the cord clamping in the partograph.”

(Nurse midwife)

“There should be proper supervision and monitoring also from central level or higher level.”

(Nurse midwife)

“In my experience, without follow up, without supervision and monitoring, it is impossible to achieve 100% adaptation”

(SBA trainer/supervisor)

The participants thought using a flex chart, poster and pamphlet could be methods of raising awareness. Using media like radio, television and social media could also be added to raise awareness. These methods will not only raise awareness among delivery staff but also among the general public, which would help to encourage delivery staff to perform DCC.

“We can do advocacy, it will be effective to do it through television or radio, now everyone has Facebook.”

(Matron)

“If we can keep it as a slogan in audio, TV or other… like ‘delayed cord clamping is beneficial for baby’ then it will be more effective.”

(SBA trainer/supervisor)

“If the mother knows about it through the media, radio, then they might be reminded to delay doing it.”

(Nurse midwife)

### Opportunities for change

The participants mentioned that there are also opportunities to bring about change both within the hospitals as well as in the whole country. One such opportunity is the inherent will to do good among health-care staff. This provides the staff with self-motivation and it was believed that this would make it easy to bring about change. The participants displayed the understanding that they are already working for the betterment of mothers’ and children’s health and they feel happy to change their practice if it is good for mother and child.

“We conduct delivery with the good health of mother and baby in mind, therefore, if delayed cord clamping helps the baby then we will do it for the benefits it provides. This is how I get motivated. “

(Nurse midwife)

“Everyone accepts good things”

(SBA trainer/supervisor)

Another opportunity for change put forward was the staff’s trust in research and the authority of scientific evidence.

“We have to accept this for scientific reasons”

(Ward in-charge)

“We told them that many studies have shown benefits”

(Matron)

However, it was strongly emphasized that to implement research findings it should be approved by a higher authority, either at government or hospital level, providing instructions for the change. The power of hierarchical structures was pointed out, not only in relation to protocol development (need to bring to bear uniformity), but also as an opportunity to implement change through authority and direction.

“If higher authority like the in-charge, if she says, ‘you have to do it and if you don’t do it then action will be taken’, then they might do it”

(Senior nurse midwife)

“It should be the hospital director, then after that the matron and then supervisors and then after that the in-charge, and then after the in-charge all the duty station staff who should receive information and should tell them its advantages.”

(Matron)

“It should go through the Ministry, the Nepal Government. The Nepal Government is the valid authority”

(Matron)

## Discussion

All participants had heard about DCC, but not all were aware of its benefits for the newborn. All thought that DCC is easy to perform and that it does not need additional skills, efforts, equipment or manpower, since it is only a question of time to wait for a few more minutes before clamping the cord. ECC is however a deeply rooted practice in maternity care and changing this practice requires much more than changing the perceptions [9]. Evidence from a recent study in Nepal stated that DCC provides a sufficient amount of iron to reduce iron deficiency for up to eight months and reduce anemia for up to 12 months after birth [2] and has positive effects on neurodevelopment at 12 months of age in children born at term [3]. Despite this clear evidence of the benefits, results indicate that delivery care staff faces constraints when trying to implement DCC. Medical conditions and the physical environment sometimes prompt early clamping and staff tends to fall back into old routines and habits. Lack of structures and authorized protocols and instructions, together with lack of coherence in the team prevent the effective implementation of a new practice. This is in line with a study from USA that concluded that obstetricians’ beliefs about the appropriate timing for cord clamping are not consistent despite that multiple randomized controlled trials have demonstrated the beneficial impacts of DCC on neonatal outcomes [14]. Another multi-country study stated that obstetricians had difficulties in implementing DCC in clinical practice and expressed the need to produce guidelines from the Royal College of Obstetricians and Gynecologists to bring DCC into obstetric practice [15]. Yet another study mentioned the interpersonal variation in cord clamping practice and therefore suggested clear practice guidelines for the optimal time of cord clamping, taking into consideration the health benefits of DCC in both term and preterm infants [12].

The participants in this study felt that it was difficult to implement a new practice due to their regular habitual practice which made them do what they were accustomed to doing. They highlighted that regular practice can help to quickly bring about change. It is known that health-care providers do not change their practice in a single, defined moment of time; it generally occurs gradually over time when the practice is being repeated many times [16]. This is something that health system leadership needs to relate to and continuous efforts are needed, not only one-off directives. Furthermore, in the absence of refresher trainings and communication of new routines, staff is forced to rely on informal learning, listening to rumors and picking up new evidence from various channels. This creates a lack of coherence within the health system and reduces its effectiveness. Findings from this study highlight the need to improve governance within the health system and to increase the understanding of the difficulties of implementation. Health-care staff should not be left to their own devices to introduce new routines and change practices. The will to do good was presented as an opportunity for change and paired with a high level of trust in research evidence, change can be achieved if combined with a top-down approach. Simulation in combination with institutional policy has for example been proven to achieve change of practice in India [17].

This study also has some limitations worth mentioning. Due to limited time and resources, birth attendants from all the birth centers could not be included in the study. There is also always a risk of social desirability bias in a study like this, especially in a hierarchical system like Nepal. Actual perceptions and attitudes may therefore vary from self-reported, just as it is not always certain that reported practices are the actual practices in force. Efforts to create a safe space for the interviews and guarantee anonymity of results were taken. Supervisors and other persons of authority were not present during discussions. We undertook several measures to ensure trustworthiness as recommended in qualitative research [13].

### Credibility

To achieve credibility, the participants in the study i.e. delivery care staff, ward in-charges and SBA supervisors/trainers who have experiences of the phenomenon under study and are able to speak about it were included in the discussions. The questions used in the discussions were open-ended to allow for different and distinct views from the participants. The discussions continued until all the topics in the guide were covered and all the participants had been able to state their thoughts, thus indicating saturation of the data. Despite the low number of participants in each FGD, the topic was covered from many different perspectives, providing a rich variety of opinions and information. The potential problem of hierarchy was reduced by allowing the ward in-charges and SBA supervisors to form a separate group. All the participants had the opportunity to express their views. All statements from the interviewees were given equal consideration. Using the same moderator for all FGDs and KIIs ensured that the topics were covered in a consistent way.

### Transferability

We formed eight focus groups and 12 KIIs. In the FGDs, there were 3–5 participants in each group. Ideally, there would have been 5–10 in each group [18]. The sampling process of using the wards to form each group was defined as purposive, but the availability of nurse midwives who were on-duty created a limitation in the selection of staff. The FGDs and KIIs used two different levels of participants, i.e. the FGDs with delivery staff and KIIs with the ward in-charges and SBA trainers who were supervisors for the whole unit, which was assessed as the best method for data collection. The selection of participants was also important for the transferability of the findings together with an accurate and rich description of the context of the study.

### Credibility and authenticity

Having good contextual knowledge is valuable in qualitative research. The moderator (first author) was familiar with the health-care settings where the research was conducted. When analyzing data, effort should be made to stay as neutral as possible, but interpretation of the text in qualitative research always provides multiple meanings and researchers might influence the results through their personal history and pre-understanding [13]. To reduce this risk all the authors were involved in the work to determine the categories so as to create a consensus on the findings, where the second and last author provided an outside perspective on the material.

## Conclusion

The participants of this study demonstrated positive attitudes towards DCC as it was perceived as an easy task with good benefits for mother and child. However, delivery staff is left unsupported by management and has to rely on their own decisions and skills development, which makes staff hesitant to apply delayed cord clamping despite the perceived benefits. In order to change cord clamping practices to align with evidence and policy, health staff needs to be better supported by the governance structures of the health system, with clear and approved guidelines and coherent training and support made available.

## Supporting information

S1 File Interview guideline in Nepali. (PDF)

S2 File Interview guideline in English. (PDF)

S3 File Informed consent form in Nepali. (PDF)

S4 File Informed consent form in English. (PDF)

## Abbreviations

DCC: Delayed cord clamping
ECC: Early cord clamping
FGD: Focus Group Discussion
KII: Key Informant Interview
MNSC: Maternal and Neonatal Service Center
PMWH: Paropakar Maternity and Women’s Hospital
SBA: Skilled Birth Attendant
TUTH: Tribhuvan University Teaching Hospital
