# Supplementary material for: Implementing delayed umbilical cord clamping in Nepal—Delivery care staff’s perceptions and attitudes towards changes in practice
Source: PLoS One. 2019 Jun 12;14(6):e0218031. doi: 10.1371/journal.pone.0218031 (PMC6561554; doi:10.1371/journal.pone.0218031)
Supplement: S1 File — (PDF) [file pone.0218031.s001.pdf]

अनुसूचि १

लक्षित समूह छलफल निर्देशिका

१ तपाईंलाई नाभि काट्ने नयाँ प्रकृयाको बारेमा थाहा छ? थाहा छ भने कसरी थाहा पाउनु भएको हो ?

२. तपाईं साधारणतया बच्चा जन्मिएको कति समय पछि नाभि काट्नुहुन्छ?

३. महिना पुगेर जन्मेको बच्चाको नाभि ढिलो काट्ने सम्बन्धमा तपाईंको के धारणा छ? कृपया विस्तारमा भनिदिनुहोस् ।

४. तपाईंको विचारमा ढिलो नाभि काट्न आमा वा बच्चाको तर्फबाट के कस्ता अवरोधहरु आउन सक्छन्?

५. तपाईंको विचारमा ढिलो नाभि काट्न कस्ता खालका व्यक्तिगत वा व्यवसायिक अवरोधहरु आउन सक्छन्?

६. ढिलो नाभि काट्ने प्रकृया अवलम्बन गर्न तपाईंलाई के कुराले सहयोग वा प्रोत्साहन गर्छ ?

७. के तपाईंलाई तपाईंको संस्थामा ढिलो नाभि काट्ने सम्बन्धी तथ्यमा आधारित प्रोटोकलको आवश्यकता छ भन्ने लाग्छ? यसले गर्भवती महिलाको छनौट, गर्भ उमेरको मूल्यान्कन, प्रसूतिको अग्रिम तयारी तथा आमाबुवाको लागि शिक्षा जस्ता पक्षहरुलाई समेट्दछ ।

अनुसूचि २

प्रमुख सूचनादाता अर्न्तवार्ता निर्देशिका

१. महिना पुगरे जन्मेको बच्चाको नाभि ढिलो काट्ने सम्बन्धमा तपाईंको धारणा के छ? कृपया विस्तारमा भनिदिनुहोस् ।

२. तपाईंको विचारमा ढिलो नाभि काट्न आमा वा बच्चाको तर्फबाट के कस्ता अवरोधहरु आउन सक्छन्?

३. तपाईंको विचारमा ढिलो नाभि काट्न कस्ता खालका व्यक्तिगत वा व्यवसायिक अवरोधहरु आउन सक्छन्?

४. ढिलो नाभि काट्ने प्रकृया अवलम्बन गर्न तपाईंलाई के कुराले सहयोग वा प्रोत्साहन गर्छ ?

५. तपाईंको विचारमा उत्तम व्यवहारहलाई लागु गर्नका लागि प्रभावकारी परिवर्तन व्यवस्थापनका रणनीतिहरु के होलान् ? विगतमा तथ्यमा आधारित चिकित्सा अभ्यास लागु गर्ने अनुमती पाउने क्रममा तपाईंको हकमा उपयोगी सिद्ध भएका परिवर्तन व्यवस्थापनका रणनीतिहरु के के छन् ?
