# Supplementary material for: Implementing delayed umbilical cord clamping in Nepal—Delivery care staff’s perceptions and attitudes towards changes in practice
Source: PLoS One. 2019 Jun 12;14(6):e0218031. doi: 10.1371/journal.pone.0218031 (PMC6561554; doi:10.1371/journal.pone.0218031)
Supplement: S2 File — (PDF) [file pone.0218031.s002.pdf]

Annex I

FGD guideline

1. What is recent strategy to clamp the cord in managing the third stage of labor? How did you know this?
2. When do you clamp the cord as a strategy for managing the third stage of labor in normal routine? Approximate timing
3. What are your thoughts on delayed cord clamping in full term infants? Can you elaborate bit more (probe)?
4. In your view, what could be maternal/fetal barriers to use delayed cord clamping?
5. What is your opinion about personal/professional barriers to use delayed cord clamping?
6. In your opinion, what can help/encourage you to utilize delayed cord clamping?
7. Do you believe there is a need for evidence-based delayed cord clamping protocol at your facility? This would include patient selection, gestational age assessment, communication tips including a possible plan prior to delivery and parent education.

Annex II

Key informant interview guideline

1. What is your opinion about delayed cord clamping in full term infants? Can you elaborate bit more (Probe)?
2. In your view, what could be maternal/fetal barriers to use delayed cord clamping?
3. In your opinion, what could be personal/professional barriers to use delayed cord clamping?
4. In your opinion, what can help/encourage birth attendants to utilize delayed cord clamping?
5. What is your opinion about effective change management strategies for implementing best practice? What are effective change management strategies that have previously worked for you to allow successful implementation of evidence based medical practice in the healthcare setting?
