# Supplementary material for: Implementing delayed umbilical cord clamping in Nepal—Delivery care staff’s perceptions and attitudes towards changes in practice
Source: PLoS One. 2019 Jun 12;14(6):e0218031. doi: 10.1371/journal.pone.0218031 (PMC6561554; doi:10.1371/journal.pone.0218031)
Supplement: S3 File — (PDF) [file pone.0218031.s003.pdf]

मन्जुरीनामा

नाभि काट्ने उपयुक्त समय सम्बन्धी अनुसन्धानमा यहाँलाई निमन्त्रण गर्न चाहन्छु । प्रसूति कक्षमा संलग्न भएको नाताले यहाँलाई यस अध्ययनका लागि छनौट गरिएको हो । यस अध्ययनमा संलग्न हुनु अगाडी कृपया यो सहमती पत्र पढी कुनै जिज्ञासा भएमा सोध्न सक्नुहेनछ ।

प्रकृया

हजुरलाई अर्न्तवार्तामा भाग लिई नाभि काट्ने विषयमा आफ्नो धारणा र दृष्टीकोण बताउनका लागि प्रश्नहरु सोधिनेछ । अर्न्तवार्ता लगभग ३० देखि ४५ मिनेटको हुनेछ र यसलाई टेप रेकर्ड गरिनेछ । अर्न्तवार्ता सकिएपछि अर्न्तवार्ताको रेकर्डलाई अनुवाद गरिनेछ र रेकर्डलाई नष्ट गरिनेछ । पहिचान गर्न नसक्ने बनाउनका लागि अर्न्तवार्तामा संलग्न नभएको व्यक्तिलाई अनुवाद गर्न लगाईनेछ ।

गोप्यता

यस अन्तवार्ताबाट प्राप्त सम्पूर्ण तथ्यांकहरु, जानकारीहरु सुरक्षित राखिनेछ र सुरक्षाको लागि ताला लगाएर राखिनेछ र त्यसमा अध्ययन टोलिको मात्र पहूँच हुनेछ । हजुरको नाम, पहिचान वा फोन नम्बर सबै गोप्य राखिनेछ । अध्ययन टोलीले सबै सूचनाहरु गोप्य राख्नेछ र कुनै पनि प्रतिवेदनमा यहाँको नाम आउने छैन ।

स्वेच्छिक सहमती

यस अध्ययनका लागि गरिने सहभागीता पूर्णरुपमा स्वैच्छिक हो । अध्ययनमा भाग लिने वा नलिने निर्णयले तपाईंको हालको वा भविश्यको रोजगारमा कुनै दक्खल पर्ने छैन । सहभागी हुन सहमत भएपश्चात पनि अर्न्तवार्ताको कुनै प्रश्नको उत्तर दिन मन नलागेमा वा कुनै पनि समयमा अर्न्तवार्ता छोड्न मन लागेमा यहाँले छोड्न सक्नुहुन्छ र यसले हाम्रो सम्बन्धमा कुनै नकारात्मक प्रभाव पार्ने छैन ।

सम्पर्क व्यक्ति

अनुसन्धानकर्ता : निशा राना

फोन नं : ९८४१५३०२२४

ई-मेल : nishaarana@gmail.com

व्यक्तिगत सूचनका लागि जिम्मेवार : डाटा सुरक्षा निगरानी समिति

सहभागी अध्ययन सहमती

मैले तपाईंले दिएको जानकारी राम्ररी पढें वा बुझें । मैले नबुझेको वा बुझ्न चाहेको वा सबै कुरा राम्ररी बुझाईयो । म यस अध्ययनमा सहभागी हुनु स्वईच्छले सहमत छु ।

सहभागीको नाम :

सहभागीको हस्ताक्षर :

मिति :

स्थान:

यस मन्जुरीनामामा हस्ताक्षर गरी यहाँ दिईएको सबै जानकारीहरु राम्ररी बुझी आफ्नो सहमती दिएको प्रमाणित गर्दछु ।
