# Supplementary material for: Implementing delayed umbilical cord clamping in Nepal—Delivery care staff’s perceptions and attitudes towards changes in practice
Source: PLoS One. 2019 Jun 12;14(6):e0218031. doi: 10.1371/journal.pone.0218031 (PMC6561554; doi:10.1371/journal.pone.0218031)
Supplement: S4 File — (PDF) [file pone.0218031.s004.pdf]

Consent form

You are invited to be in a research study of timing of umbilical cord clamping. You were selected as a participant because you are part of the delivery healthcare team. Please read this form and ask any questions you may have before agreeing to be in the study.

Procedures:

You will be asked to participate in interview to answer questions regarding perception and attitude toward umbilical cord clamping. The interview will be around 30-45 minutes in length and it will be tape recorded. After completion of the interview, the tape recordings will be transcribed and the audio tapes will be destroyed. A person who is not involved in interview/ discussion will be assigned to transcribe the record there will be no identifiers.

Confidentiality:

The confidentiality of your response and information to this interview will be protected and kept locked and be accessed only by the research team. Your name, identity or phone number will not be used. Research team will protect your information to the best ability. You will not be named in any reports.

Voluntary participation

Participation in this study is voluntary. Your decision whether or not to participate will not affect your current or future relations and your employment status. If you decide to participate, you are free to not answer any question or withdraw at any time without affecting those relationships.

Contact person:

Researchers: Nisha Rana
Phone number :9841530224
e-mail addresses: nishaarana@gmail.com
The body responsible for personal data: Data Safety Monitoring Committee

I have read the foregoing information, or it has been read to me. I have had the opportunity to ask questions about it and any questions that I have asked have been answered to my satisfaction. I consent voluntarily to participate as a participant in this study.

Name of Participant: ____________________

Signature of Participant: ____________________

Date: ____________________ (dd/mm/yy)

Place: ____________________

By signing this I certify that I have read the information and agree to participate in the study.
